# Ultrasensitive circular dichroism spectroscopy based on coupled quasi-bound states in the continuum

**DOI:** 10.1515/nanoph-2024-0620

**Published:** 2025-01-17

**Authors:** Tingting Guan, Zhenyu Wang, Ruize Wang, Zihan Wu, Chaowei Wang, Dong Wu, Jiaru Chu, Yang Chen

**Affiliations:** Chinese Academy of Sciences Key Laboratory of Mechanical Behavior and Design of Materials, Department of Precision Machinery and Precision Instrumentation, 12652 University of Science and Technology of China, 230027 Hefei, China

**Keywords:** CD spectroscopy, chiral sensing, mode coupling, bound state in the continuum

## Abstract

Circular dichroism (CD) spectroscopy is essential for biochemistry, structural biology and pharmaceutical chemistry. While the chiroptical properties of chiral molecules are characterized by the Pasteur parameter *κ*, it is commonly conceived that the generation of CD is solely attributed to the imaginary part *κ*′′. However, since the imaginary part *κ*′′ is orders of magnitude smaller than the real part *κ*′ for most chiral molecules, the achievable sensitivity of CD spectroscopy is quite limited. Here, we report a recipe for realizing ultrasensitive CD spectroscopy based on the *κ*′ component of chiral molecules. Two quasi-bound states in the continuum are coupled by chiral molecules to form two hybridized branches, whose wavelengths and eigenpolarizations are very sensitive to the value of *κ*′. Giant CD signals over four orders of magnitude larger than the case without mode coupling are thus produced, paving the way towards chiral structure analysis at the single molecule level.

## 1 Introduction

Chirality is essential for life [1]. An overwhelming majority of chemically and biologically active molecules are chiral, such as proteins, DNAs and sugars. Circular dichroism (CD) spectroscopy is a fundamental tool for investigating chiral molecules [2], [3], [4], [5], which can be harnessed for deriving the structural information of molecules and discriminating chiral molecules of opposite handedness, *i.e.*, enantiomers. It measures the difference in absorption of right- and left-handed circularly polarized (RCP and LCP) light by chiral molecules. Generally, the chiroptical responses of molecules are characterized by the complex-valued Pasteur parameter *κ* = *κ*′ + *iκ*′′, and only the imaginary part *κ*′′ is responsible for the generation of CD signals [4], [5]. However, while the *κ* value of natural chiral molecules is typically quite small due to the scale mismatch between the light wavelength and the molecular wavefunction, their imaginary part *κ*′′ is even much smaller than the real part *κ*′ (*κ*″ ≪ *κ*′) [6], [7], resulting in the obtained CD signals extremely weak. In order to amplify CD signals to a measurable level, molecule solutions with high concentration and large volume have to be prepared, which inherently limits the sensitivity of CD spectroscopy to achieve the ultimate goal of single molecule detection.

Recently, it is uncovered that the amplitude of CD is also related to the optical helicity density at the location of the molecule, which is quantified by $C=\frac{\varepsilon_{0}\omega }{2}\cdot Im\left(E^{*}\cdot B\right)$ [8]. A general equation is thus derived: $CD=A_{R}-A_{L}=\frac{4}{\varepsilon_{0}}\kappa \prime \prime \cdot C$ , where *A*_*R*_ and *A*_*L*_ are the absorption of RCP and LCP light, and *ɛ*_0_ is the permittivity of free space. As guided by this relationship, artificial nanostructures supporting superchiral fields of large *C* have been extensively proposed for boosting the sensitivity of CD spectroscopy [9], [10], [11], [12], [13], [14], [15], [16], [17], [18], [19], [20], [21], [22], [23], [24], but their achievable sensitivity is still limited by the extremely small value of *κ*″. On the other hand, although the real part *κ*′ of chiral molecules can be over two orders of magnitude larger than *κ*″, it is still unclear how to harness *κ*′ for realizing sensitive CD spectroscopy.

In this work, we demonstrate a new strategy to achieve ultrasensitive CD spectroscopy based on the real component *κ*′, rather than the imaginary component *κ*′′. The physics of bound state in the continuum (BIC), that has recently been utilized in photonics for realizing and engineering high-*Q* resonances [25], [26], [27], [28], [29], [30], is harnessed as a workhorse for enhancing chiral light–matter interactions. Through delicate design of a photonic crystal slab (PCS), two quasi-BICs are coupled by chiral molecules to form two hybridized branches, whose wavelengths and eigenpolarizations are strongly dependent on the value of *κ*′. Giant CD signals over four orders of magnitude larger than the case without the PCS are then produced, which has never been realized before and can serve as an ultrasensitive tool for analyzing enantiomers.

## 2 Paradigmatic system design

**Figure 1:**
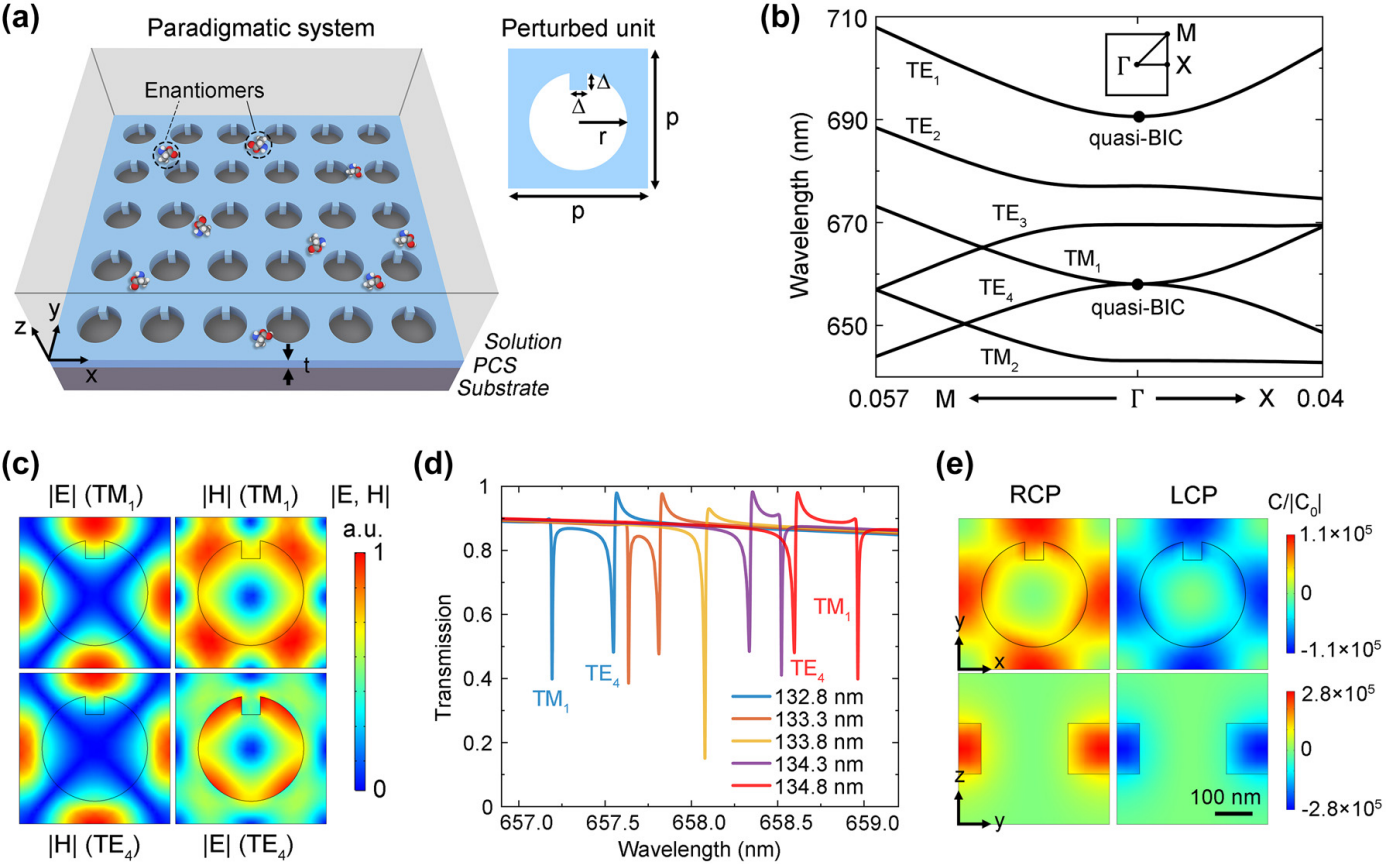
Metasurface design. (a) Schematic of the paradigmatic system for *κ*′-based CD spectroscopy. The geometric parameters are: period *p* = 400 nm, radius *r* = 140 nm, perturbation Δ = 50 nm, and thickness *t* = 133.8 nm. (b) Calculated bandstructure of the PCS with a series of quasi-BICs occurring at the *Γ* point. (c) Electric and magnetic field distributions of the TM_1_ and TE_4_ quasi-BICs exhibiting electromagnetic duality. (d) Simulated transmission spectra of the PCS under RCP incidence for different slab thickness *t*. (e) Simulated optical helicity density *C* distributions of the PCS (*t* = 133.8 nm) under RCP (left) and LCP (right) incidence at the cross-sectional *x*-*y* plane (top) and *y*-*z* plane (bottom), which are normalized by the reference *C* value of free-space RCP light.

Figure 1a illustrates a paradigmatic system for leveraging the real component *κ*′ of chiral molecules in CD spectroscopy. A PCS composed of arrayed nanoholes inside a dielectric slab (n = 2.3) is positioned on a substrate (n = 1.45), and covered by a solution with a permittivity of *ɛ*_*r*_ = 2.1 + 9*i* × 10^−5^ and a permeability of *μ*_*r*_ = 1 + 4*i* × 10^−5^. A set of design principles are implemented. First, the structure itself should be achiral to make sure that the acquired CD signals are exclusively originated from chiral molecules, without the disturbance from the CD responses of chiral structures. Second, the associated resonances should be helicity-preserved over the volume. Otherwise, CD signals coming from different locations could be oppositely signed and cancel each other in the far field. Third, high-*Q* resonances with strong field enhancement and localization are desired to enhance light–matter interactions.

With respect to our proposed PCS, perturbative notches *Δ* are introduced to break the $C_{2}^{z}$ symmetry of circular nanoholes, so that those symmetry-protected nonradiative BICs at the *Γ* point are converted to high-*Q* quasi-BICs (Figure 1b). Meanwhile, the structure still maintains the mirror symmetry of *y*-*z* plane to be achiral. To construct helicity-preserved resonances, the TM_1_ and TE_4_ quasi-BICs are utilized for their electromagnetic duality [20], [31], meaning that the electric field distributions of TM_1_ mode resemble the magnetic field distributions of TE_4_ mode and *vice versa* (Figure 1c). Then, these two modes are made degenerate at the *Γ* point by finely tuning the thickness of PCS, which can be realized in fabrication through atomic layer deposition (ALD). We simulate the transmission spectra of the PCS under normal incidence of RCP light for different thickness *t*, as shown in Figure 1d. two sharp resonances are observed for *t* = 132.8 nm, corresponding to TM_1_ and TE_4_ quasi-BICs, respectively. When the thickness *t* is increased, the two resonances approach each other, merge into one dip, and then separate again, but their relative positions are exchanged. For the case of degeneracy at *t* = 133.8 nm, the optical helicity density *C* in the near field maintains positive and negative for RCP and LCP incidence respectively (Figure 1e), validating the realization of helicity preservation. Moreover, the absolute value of *C* can be enhanced by over five orders of magnitude when compared to that of free-space circularly polarized light, which lays the foundation for strong chiral light–matter interactions.

## 3 Results of CD spectroscopy

**Figure 2:**
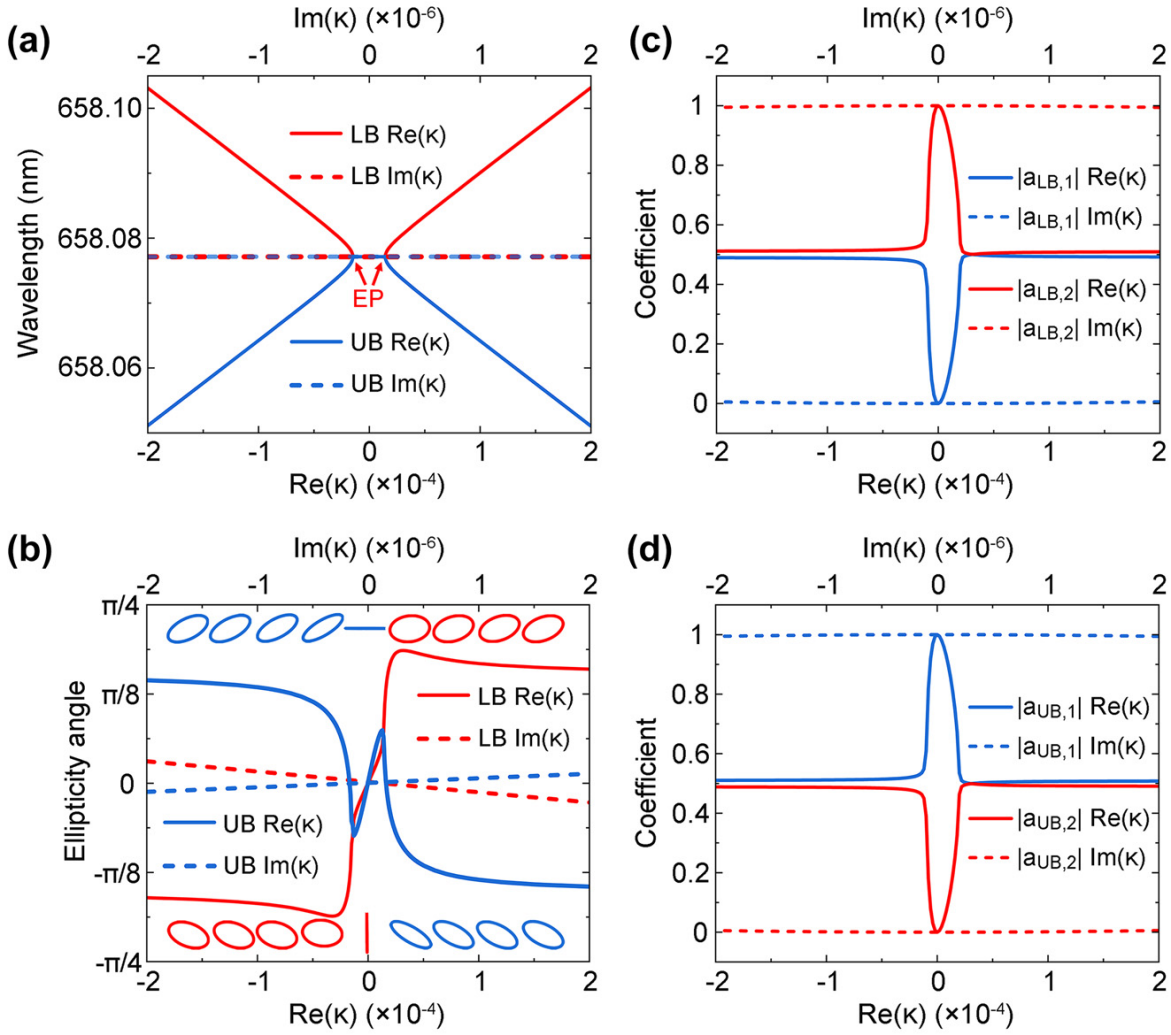
Coupling between two bound states in the continuum. (a, b) Wavelengths of UB and LB (a) and their radiative elliptical angles (b) as functions of the real and imaginary part of *κ*, which are normalized by *κ*_0_. The evolution of radiative eigenpolarizations is indicated in (b), where the red color and blue color indicate right-handed and left-handed elliptical polarization, respectively. (c, d) Retrieved coupling coefficients *a*_*j*_ for LB (c) and UB (d) as functions of *κ*, where the contributions of real and imaginary part are considered separately. *a*_1(2)_ corresponds to TM_1_ (TE_4_) quasi-BIC.

Next, we introduce chiral molecules into the solution to enable a nonzero *κ*. The initial Pasteur parameter is set to $\kappa_{0}=\left(2+0.02\right)\times 10^{-4}$ , exhibiting a realistically low magnitude and containing a typical ratio between the real and imaginary part [6], [7]. The nonzero *κ* induces magnetoelectric interactions in the Maxwell equations, so that the two originally orthogonal modes, TM_1_ and TE_4_ quasi-BICs, can now couple with each other, forming two hybridized branches, *i.e.*, upper branch (UB) and lower branch (LB). The Hamiltonian H of the coupled system is written as [14]:

(1) $$H=\left[\begin{array}{cc} \omega_{1} & iA\kappa \omega_{2}\\ iA\kappa \omega_{1} & \omega_{2} \end{array}\right],$$

where *ω*_1_ and *ω*_2_ are the frequencies of TM_1_ and TE_4_ quasi-BICs, *A* is a real-valued parameter associated with the field overlapping of the two modes. In our case, since *κ*″ ≪ *κ*′, Eq. (1) can be approximated to:

(2) $$H=\left[\begin{array}{cc} \omega_{1} & iA\kappa \prime \omega_{2}\\ iA\kappa \prime \omega_{1} & \omega_{2} \end{array}\right],$$

which exhibits a typical anti-parity-time (anti-PT) symmetry at zero detuning *ω*_1_ = *ω*_2_. As a result, the two hybridized resonances possess the same wavelength before the exceptional point (EP), as shown in Figure 2a. Beyond the EP, their wavelengths are split and approximately follow linear relationships with *κ*′, while almost have no dependence on the value of *κ*″.

Along with Rabi splitting is the modulation of radiative polarization. When no coupling is involved for *κ* = 0, the TM_1_ and TE_4_ quasi-BICs possess linear eigenpolarizations along *x*- and *y*-direction, respectively. Once enantiomers with positive *κ* are introduced, optical chirality emerges in the two hybridized branches, whose evolution can be divided into two stages as shown in Figure 2b. Initially, the ellipticity angles of both branches are rapidly increased to enable right-handed elliptical polarizations. But beyond the EP, the two branches experience distinct changes: while the ellipticity angle of LB continues to rise steeply and then undergoes a gradual decrease after the saturation point, that of UB rapidly declines to be left-handed elliptically polarized and then converts to a gradual decrease. If a negative *κ* is introduced instead, the evolution processes of ellipticity angles are exchanged for LB and UB. Meanwhile, as clearly revealed in Figure 2b, it is the real part *κ*′ that has substantial influence on the radiative polarization, and the impact of *κ*′′ is much weaker.

To further analyze the chirality-induced mode hybridization, UB and LB are written as the superpositions of TM_1_ and TE_4_ quasi-BICs, where the coefficient *a*_*j*_ basically indicates the strength of hybridization. As depicted in Figure 2c, mode hybridization is initiated by the introduction of a nonzero *κ*′ and rapidly promoted, with the proportion of TM_1_ quasi-BIC among LB declining from 1 to 0.5, while that of TE_4_ quasi-BIC growing from 0 to 0.5. This process is accomplished at the EP, beyond which *a*_LB, 1_ and *a*_LB, 2_ maintain around 0.5. If only the imaginary part *κ*′′ is involved, mode hybridization will not happen, as suggested by the flat dashed lines of *a*_LB, 1_ and *a*_LB, 2_ in Figure 2c. The dependence of coefficients on *κ* is complementary for UB as shown in Figure 2d.

**Figure 3:**
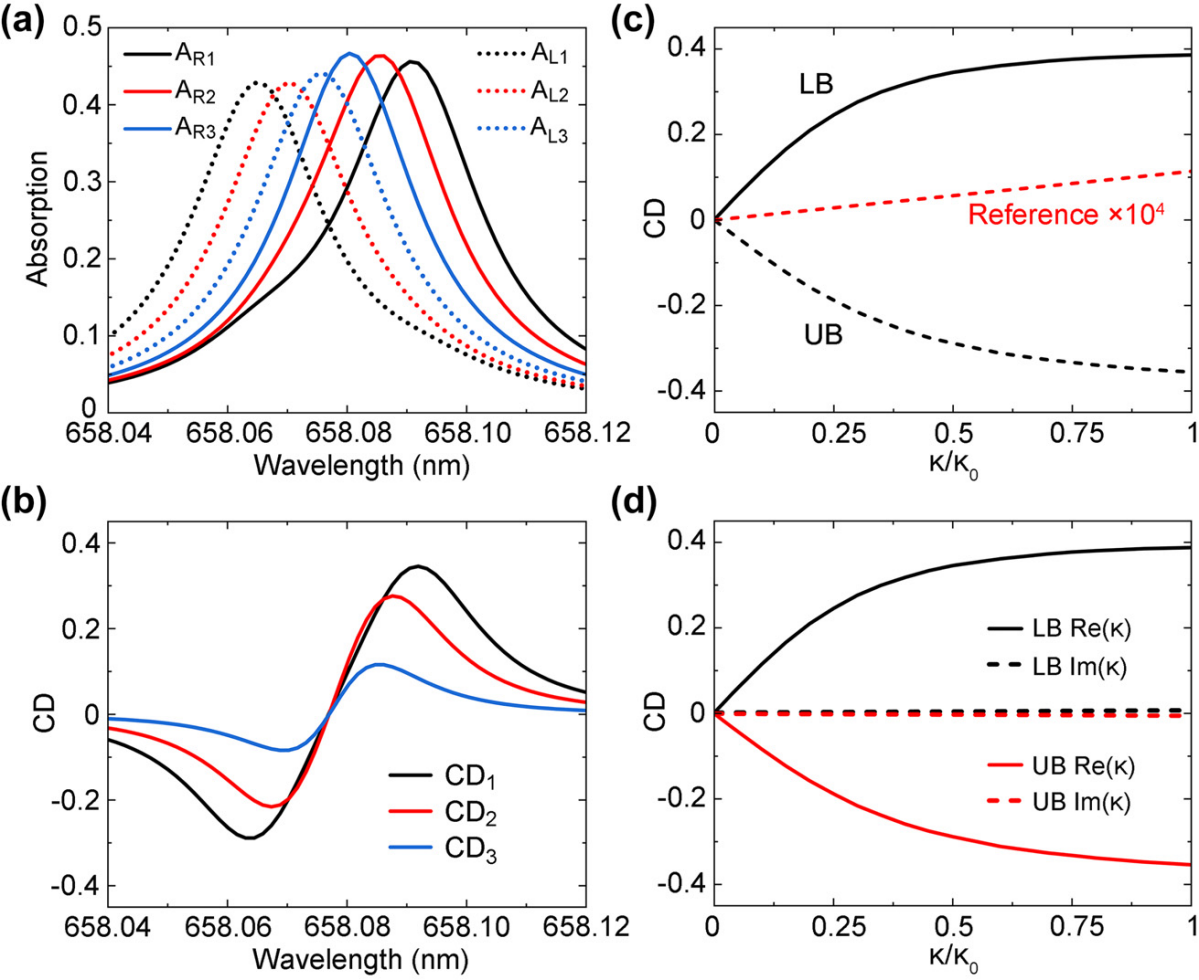
Enhanced circular dichroism. (a, b) Simulated absorption spectra of the PCS under RCP and LCP incidence for different *κ* values (a) and the corresponding CD spectra (b). The subscript 1, 2, 3 corresponds to the *κ* value of 0.5*κ*_0_, 0.3*κ*_0_ and 0.1*κ*_0_, respectively. (c) Retrieved CD values of LB and UB as a function of *κ*. The reference case of enantiomers without the PCS is multiplied by four orders of magnitude for comparison. (d) Dependence of CD values on *κ* if only the real part *κ*′ or imaginary part *κ*′′ is considered.

In Figure 3a, we simulate the absorption spectra of the PCS for different *κ* under RCP and LCP incidence. For a relatively large *κ* = 0.5*κ*_0_, the sharp resonances at 658.09 nm and 658.06 nm correspond to the LB and UB, respectively, which are fully separated. Since the ellipticity angles of the two branches have large absolute values but opposite signs, the RCP absorption is much larger than the LCP absorption at 658.09 nm, leading to a large CD of 0.346 (Figure 3b). Here, we define CD values as the differential absorption of the system:*CD* = *A*_*R*_ − *A*_*L*_, where *A*_*R*_ and *A*_*L*_ represent the absorption of RCP and LCP light, respectively. If *κ* is decreased, corresponding to a lower concentration of enantiomers in the solution, the resonant absorption of RCP and LCP does not change much, but their spectral positions get closer due to a smaller Rabi splitting, resulting in a larger overlap of their absorption peak. As a consequence, the acquired CD signal rapidly decreases (Figure 3b). Moreover, our retrieved CD spectra exhibit a characterized bisignate lineshape, owing to the oppositely signed ellipticity angles of the two branches.

To evaluate the sensitivity of our proposed CD spectroscopy, we plot the CD signals of UB and LB as a function of *κ* (Figure 3c). The CD amplitudes of UB and LB correspond to the dip and peak values in Figure 3b, respectively. When compared to the reference case without the PCS, the sensitivity can be boosted by over four orders of magnitude, which has never been realized before. If the contributions of *κ*′ and *κ*′′ are separately considered (Figure 3d), it is apparent that our method is primarily based on the real part. Furthermore, as shown in Figure 3c, the variation tendency of CD is nonlinear, which is fast initially and then gradually slowed down. Therefore, this method is very suitable for the trace detection of enantiomers with an extremely low concentration. Also, we notice that the initial stage with a larger slope, corresponding a higher sensitivity, is around the EP. While EPs has been well investigated for significantly enhancing the performance of refractive sensing [32], [33], it is the first time to our knowledge that the physics of EP has shown great potential in ultrasensitive chiral sensing.

## 4 Discussion and conclusion

**Figure 4:**
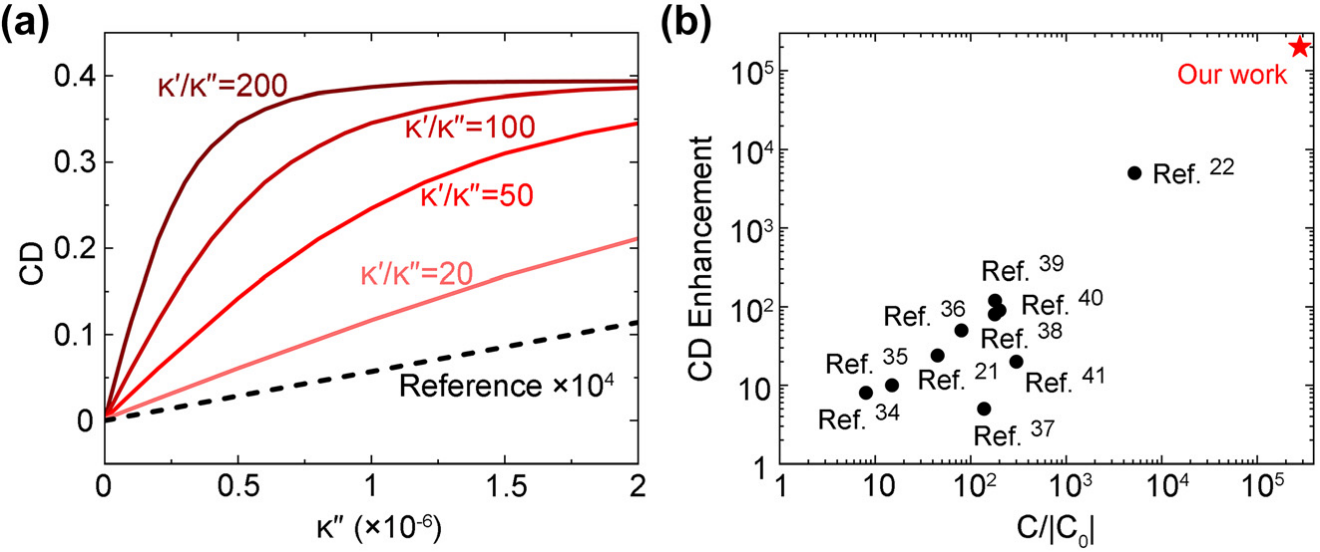
Benckmark of our approach. (a) Retrieved CD values as a function of *κ*″, where different ratios *κ*′/*κ*″ are discussed. (b) Comparison between our approach and some typical chiral sensing works based on superchiral nanostructures in terms of the enhancement of CD and optical helicity density C.

Although the relationship of *κ*″ ≪ *κ*′ is general for natural chiral molecules, their particular ratio depends on the type of molecule. In Figure 4a, we discuss the dependence of CD on *κ*″ for different values of *κ*′/*κ*″. The amplitude of CD, along with the sensitivity of CD spectroscopy, is strongly enhanced for a larger ratio *κ*′/*κ*″, further confirming the dominant role of *κ*′ in the generation CD. Besides, even for a relatively low ratio of *κ*′/*κ*″ = 20, the sensitivity of CD spectroscopy is still boosted by four orders of magnitude, relative to the reference case, suggesting that our method can be applied for a wide variety of chiral molecules.

In Figure 4b, we compare our approach with some typical chiral sensing works based on superchiral nanostructures [21], [22], [34], [35], [36], [37], [38], [39], [40], [41], in terms of two figure of merit (FOM): enhancement of CD and optical helicity density C. Our approach is superior in both FOMs, offering unprecedented sensitivity in CD spectroscopy. Such improvement in performance is mainly attributed to two factors. First, while other approaches mainly depend on the imaginary part *κ*″, our method relies on the real part *κ*′, which is inherently two orders of magnitude larger that *κ*″. In fact, the real part *κ*′ is even ignored in some previous works [19], [20], [21], due to the conception that *κ*′ has no impact on CD. Second, the quasi-BICs we utilize for mode coupling possess high *Q*-factors and hence enable strong chiral light–matter interactions.

In conclusion, our results provide a distinct paradigm to harness the real part *κ*′ of enantiomers, but not the imaginary part *κ*″, for ultrasensitive CD spectroscopy. The coupling between two quasi-BICs with electromagnetic duality offers the platform, where *κ*′ could come into play in the generation of CD through Rabi splitting and mode chirality arising. Besides, as empowered by the physics of EP, the CD signals are very sensitive to the value of *κ*′, leading to the sensitivity of CD spectroscopy over four orders of magnitude larger than the case without mode coupling. This paves the way towards ultrasensitive detection, discrimination and structural analysis of chiral molecules, even to the single molecule level.

## 5 Simulation methods

All the simulations in this work are conducted by the finite element method solver in COMSOL Multiphysics. Bloch boundary conditions are applied in the *x* and *y* directions, while perfectly matched layers are employed in the *z* direction. To account for chiral media, the built-in electromagnetic equations have to rewritten to include the Pasteur parameter *κ*. In particular, the equations for displacement fields are modified as:

$$\begin{array}{ll} ewfd\cdot Dx & =epsilon0\text{\_}const*ewfd\cdot Ex\\ & \;+ewfd.Px-1i*\kappa *ewfd.Hx/c0, \end{array}$$

$$\begin{array}{ll} ewfd\cdot Dy & =epsilon0\text{\_}const*ewfd\cdot Ey+ewfd.Py\\ & \;-1i*\kappa *ewfd.Hy/c0, \end{array}$$

$$\begin{array}{ll} ewfd\cdot Dz & =epsilon0\text{\_}const*ewfd\cdot Ez+ewfd.Pz\\ & \;-1i*\kappa *ewfd.\;Hz/c0, \end{array}$$

The constitutive relations for ***H*** and *d* ***H***/*dt* are modified as:

$$\begin{array}{ll} ewfd\cdot Hx & =\left(ewfd\cdot murinvxx*\left(ewfd.Bx\right.\right.\\ & \;\left.-1i*\kappa *ewfd.Ex/c0\right)+ewfd.murinvxy\\ & \;*\left(ewfd.By-1i*\kappa *ewfd.Ey/c0\right)\\ & \;+ewfd.murinvxz*\left(ewfd.Bz\right.\\ & \;\left.\left.-1i*\kappa *ewfd.Ez/c0\right)\right)/mu0\text{\_}\mathrm{const}, \end{array}$$

$$\begin{array}{ll} ewfd\cdot Hy & =\left(ewfd\cdot murinvyx*\left(ewfd.Bx-1i*\kappa \right.\right.\\ & \;\left.*ewfd.Ex/c0\right)+ewfd.murinvyy\\ & \;*\left(ewfd.By-1i*\kappa *ewfd.Ey/c0\right)\\ & \;+ewfd.murinvyz*\left(ewfd.Bz\right.\\ & \;\left.\left.-1i*\kappa *ewfd.Ez/c0\right)\right)/mu0\text{\_}\mathrm{const}, \end{array}$$

$$\begin{array}{ll} ewfd\cdot Hz & =\left(ewfd\cdot murinvzx*\left(ewfd.Bx-1i*\kappa \right.\right.\\ & \;\left.*ewfd.Ex/c0\right)+ewfd.murinvzy\\ & \;*\left(ewfd.By-1i*\kappa *ewfd.Ey/c0\right)\\ & \;+ewfd.murinvzz*\left(ewfd.Bz-1i*\kappa \right.\\ & \;\left.\left.*ewfd.Ez/c0\right)\right)/mu0\text{\_}\mathrm{const}, \end{array}$$

$$\begin{array}{ll} ewfd\cdot dHdtx & =\left(ewfd\cdot murinvxx*\left(ewfd.dBdtx-1i*\kappa \right.\right.\\ & \;\left.*ewfd.iomega*ewfd.Ex/c0\right)\\ & \;+ewfd.murinvxy*\left(ewfd.dBdty\right.\\ & \;\left.-1i*\kappa *ewfd.iomega*ewfd.Ey/c0\right)\\ & \;+ewfd.murinvxz*\left(ewfd.dBdtz\right.\\ & \;-1i*\kappa *ewfd.iomega\\ & \;\left.\left.*ewfd.Ez/c0\right)\right)/mu0\text{\_}\mathrm{const}, \end{array}$$

$$\begin{array}{ll} ewfd\cdot dHdty & =\left(ewfd\cdot murinvyx*\left(ewfd.dBdtx-1i*\kappa \right.\right.\\ & \;\left.*ewfd.iomega*ewfd.Ex/c0\right)\\ & \;+ewfd.murinvyy*\left(ewfd.dBdty-1i*\kappa \right.\\ & \;\left.*ewfd.iomega*ewfd.Ey/c0\right)\\ & \;+ewfd.murinvyz*\left(ewfd.dBdtz\right.\\ & \;-1i*\kappa *ewfd.iomega\\ & \;\left.\left.*ewfd.Ez/c0\right)\right)/mu0\text{\_}\mathrm{const}, \end{array}$$

$$\begin{array}{ll} ewfd\cdot dHdtz & =\left(ewfd\cdot murinvzx*\left(ewfd.dBdtx-1i*\kappa \right.\right.\\ & \;\left.*ewfd.iomega*ewfd.Ex/c0\right)\\ & \;+ewfd.murinvzy*\left(ewfd.dBdty\right.\\ & \;\left.-1i*\kappa *ewfd.iomega*ewfd.Ey/c0\right)\\ & \;+ewfd.murinvzz*\left(ewfd.dBdtz-1i*\kappa \right.\\ & \;\left.\left.*ewfd.iomega*ewfd.Ez/c0\right)\right)/mu0\text{\_}\mathrm{const}. \end{array}$$

## Supplementary Material

Supplementary Material Details
