# Supplementary material for: Ultrasensitive circular dichroism spectroscopy based on coupled quasi-bound states in the continuum
Source: Nanophotonics. 2025 Jan 17;14(8):1083–9. doi: 10.1515/nanoph-2024-0620 (PMC12019937; doi:10.1515/nanoph-2024-0620)
Supplement: Supplementary file 1 — Supplementary Material Details [file j_nanoph-2024-0620_suppl_001.docx]

**Supporting Information for**

**Ultrasensitive circular dichroism spectroscopy based on coupled quasi-bound states in the continuum**

Tingting Guan**^†^**, Zhenyu Wang**^†^**, Ruize Wang, Zihan Wu, Chaowei Wang*, Dong Wu, Jiaru Chu, Yang Chen*

*Chinese Academy of Sciences Key Laboratory of Mechanical Behavior and Design of Materials, Department of Precision Machinery and Precision Instrumentation, University of Science and Technology of China, 230027 Hefei, China*

**^†^***These authors contributed equally: Tingting Guan, Zhenyu Wang*

**Email: chaoweiw@ustc.edu.cn, cyang_phys@ustc.edu.cn*

1. Impact of photonic crystal slab thickness on CD spectroscopy


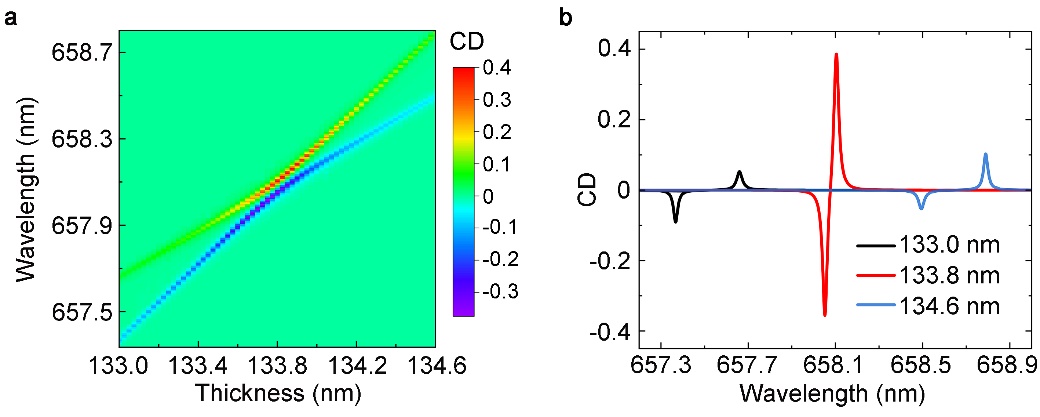


Fig. S1. (a) Simulated CD spectra of the PCS as functions of the thicknesses for a fixed κ value. (b) Simulated CD spectra for different PCS thicknesses of 133.0 nm, 133.8 nm and 134.6 nm. As revealed here, the CD spectrum is sensitive to the thickness of the PCS. Within the thickness range from 133.0 nm to 134.6 nm, the CD amplitude of the system initially increases, reaching the maximum at 133.8 nm where our system was designed to operate, and then decreases. However, even for the lower CD values (~ 0.1) at 133.0 nm and 134.6 nm, our system still demonstrates strong CD amplifications, relative to the reference case without metasurfaces. In fact, the thickness of the PCS can be precisely controlled in nanofabrication by using atomic layer deposition (ALD).

2. **Influence of different perturbation sizes on CD spectroscopy**


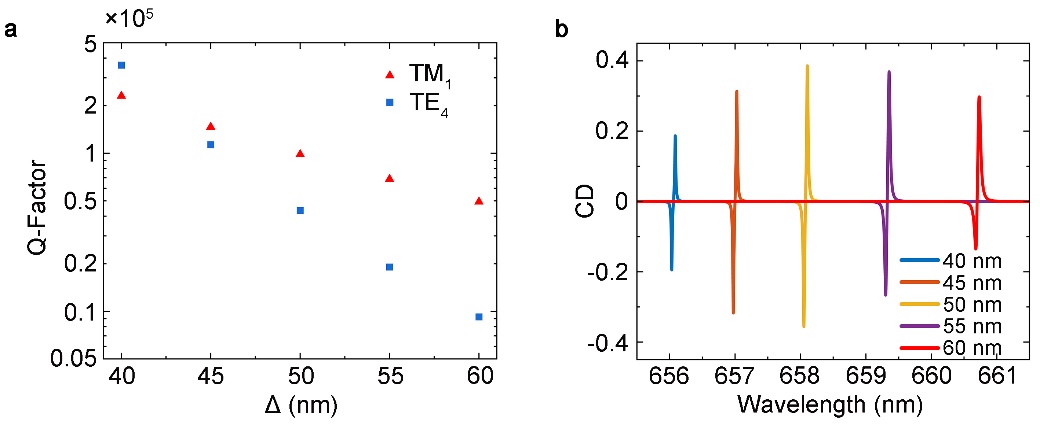


Fig. S2. (a) Simulated *Q*-factors of the TM_1_ and TE_4_ quasi-BIC mode as functions of the perturbation size Δ. (b) Simulated CD spectra of the PCS for different Δ values. As the perturbative notch size increases, the Q-factor decreases rapidly, but the CD amplitude changes slowly, initially increasing and then decreasing. Consequently, we selected a 50 nm perturbation for its ease of fabrication and strong enhancement.
